# New insights into the genetics of glioblastoma multiforme by familial exome sequencing

**DOI:** 10.18632/oncotarget.2950

**Published:** 2014-12-10

**Authors:** Christina Backes, Christian Harz, Ulrike Fischer, Jana Schmitt, Nicole Ludwig, Britt-Sabina Petersen, Sabine C. Mueller, Yoo-Jin Kim, Nadine M. Wolf, Hugo A. Katus, Benjamin Meder, Rhoikos Furtwängler, Andre Franke, Rainer Bohle, Wolfram Henn, Norbert Graf, Andreas Keller, Eckart Meese

**Affiliations:** ^1^ Clinical Bioinformatics, University of Saarland, Saarbrücken, Germany; ^2^ Institute of Human Genetics, University of Saarland, Medical School, Homburg, Germany; ^3^ Institute of Clinical Molecular Biology, Christian-Albrechts-University Kiel, Haus Niemannsweg, Kiel, Germany; ^4^ Department of Pathology, University of Saarland, Medical School, Building, Homburg, Germany; ^5^ Department of Internal Medicine III, University of Heidelberg, Heidelberg, Germany; ^6^ Pediatric Hematology and Oncology, University of Saarland, Medical School, Homburg, Germany

**Keywords:** glioblastoma multiforme, next generation sequencing, bioinformatics

## Abstract

Glioblastoma multiforme (GBM) is the most aggressive and malignant subtype of human brain tumors. While a family clustering of GBM has long been acknowledged, relevant hereditary factors still remained elusive. Exome sequencing of families offers the option to discover respective genetic factors.

We sequenced blood samples of one of the rare affected families: while both parents were healthy, both children were diagnosed with GBM. We report 85 homozygous non-synonymous single nucleotide variations (SNVs) in both siblings that were heterozygous in the parents. Beyond known key players for GBM such as ERBB2, PMS2, or CHI3L1, we identified over 50 genes that have not been associated to GBM so far. We also discovered three accumulative effects potentially adding to the tumorigenesis in the siblings: a clustering of multiple variants in single genes (e.g. PTPRB, CROCC), the aggregation of affected genes on specific molecular pathways (e.g. Focal adhesion or ECM receptor interaction) and genomic proximity (e.g. chr22.q12.2, chr1.p36.33). We found a striking accumulation of SNVs in specific genes for the daughter, who developed not only a GBM at the age of 12 years but was subsequently diagnosed with a pilocytic astrocytoma, a common acute lymphatic leukemia and a diffuse pontine glioma.

The reported variants underline the relevance of genetic predisposition and cancer development in this family and demonstrate that GBM has a complex and heterogeneous genetic background. Sequencing of other affected families will help to further narrow down the driving genetic causes for this disease.

## INTRODUCTION

Gliomas are the most common primary brain tumors in adults and are associated with a generally very dismal prognosis. The most aggressive subtype is glioblastoma multiforme (GBM) with a median survival of 15 months after surgery and subsequent treatment with temozolomide [1]. The age adjusted prognosis does not significantly differ between the two major histological subtypes, the primary GBMs that are diagnosed at an advanced stage and at a median age of approximately 60 years and the secondary GBMs that develop from preexisting low grade astrocytoma at a median age of 45 years [2]. Among brain tumors in childhood, GBM is a rather rare entity [3].

Numerous studies have focused on the genetics of this tumor to further dissect the underlying mechanisms and to contribute to a better prognosis. Over the last decade several genetic lesions including *TP53* and *PTEN* mutations have been identified in glioblastoma tissue. Recently, sequencing of over 20,000 genes discovered an extended number of altered protein coding sequences including *IDH1* (isocitrate dehydrognase) that were changed in more than 10 % of GBM [4].

Although there is no established monogenic Mendelian syndrome of heritable gliomas, there is strong epidemiologic evidence of family clustering of this tumor. Familial gliomas occur in approximately 5% of all glioma cases, the majority of which is associated with neoplastic syndromes like the Li-Fraumeni syndrome and neurofibromatosis type 1 [5]. As to the remaining familial cases, the influences of a shared environment and of the presumed hereditary components remain to be determined. Besides some cases indicative of an autosomal recessive mode of heredity, there was also anecdotal evidence for a dominant inheritance in few families [6]. Recently, a genome wide SNP linkage analysis supported the idea of a Mendelian predisposition to gliomas and specifically to a susceptibility locus at 17q12-21.32 [7].

Whole exome sequencing of affected families has been successfully applied to find genetic factors for various human pathologies, such as prostate cancer [8], Wilms tumor [9], breast cancer [10], hearing loss [11], and many other diseases. To find potential genetic factors linked to GBM tumorigenesis we performed whole exome sequencing of a rare familial case: while both parents were not diagnosed for any cancer, both siblings were diagnosed with a GBM. Following the diagnosis of a GBM the sister developed a pilocytic astrocytoma, a common acute lymphatic leukemia, and a diffuse glioma. The development of several tumors, particularly at such an early age indicates a genetic component. We sequenced germline DNA of the two affected siblings and their unaffected parents. For the two children, the GBM DNA has also been sequenced.

## RESULTS

### History of a family with two siblings with glioblastoma

**Figure 1:**
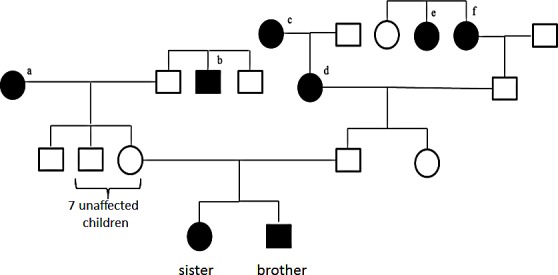
Family pedigree ^a^ melanoma at the age of 61 years, ^b^ prostate carcinoma at the age of about 50 years, ^c^ meningioma at the age of 63 years, ^d^ meningioma at the age of 86 years, ^e^ died at the age of about 50 years of ovarian cancer, ^f^ died at the age of 72 years of colon cancer.

In our study we consider a rare familial case of Glioblastoma multiforme (GBM). While both parents were healthy, two siblings were diagnosed with GBM. The brother was diagnosed at the age of 10 and the sister at the age of 12 years. The sister also developed a pilocytic infratentorial astrocytoma (WHO grade I), a common acute lymphatic leukemia and a diffuse pontine glioma. Both parents did not suffer from brain cancer or other cancer diseases. In the paternal lineage there were two ancestors with meningioma, one with ovarian carcinoma and one with colon carcinoma, each at advanced age. In the maternal lineage there were two ancestors with cancer including one with a prostate carcinoma and one with a melanoma. The pedigree is presented in Figure 1. We performed exome sequencing of germline DNA from both parents and of the siblings. Additionally, we also considered the variants in the GBM DNA of both tumors.

### Exome sequencing performance

We performed exome sequencing for the glioblastoma tissue of the siblings, their blood samples and the blood samples of their parents. Details on the tumor samples are provided in Material and Methods. All samples were sequenced on a single lane on an IlluminaHiSeq2000, yielding a total of 475 million reads. Of these, 95 % could be mapped to the human reference genome build hg19. After excluding reads that map to multiple locations, we obtained a mean coverage between 45x and 62x. Between 77 % and 83 % of the targeted sequences were covered ≥20x.

### SNV and indel analysis

To discover SNVs and indels that may be causative for GBM we called variants for all six samples together using the GATK UnifiedGenotyper [13]. We discovered a total of 100,676 different variants, of which 83,394 passed the quality filters. Next, we applied ANNOVAR [14] for annotating the genes and class of SNPs and indels. According to this annotation, 18,626 exonic SNPs and indels were non-synonymous, including missense mutations, frameshifts, gains of stop codons and losses of stop codons. First we focused on variants that were homozygous in both children's germlines while being heterozygous in both parents' germlines. We report 85 such homozygous non-synonymous SNVs, mapping to 73 different genes. The respective mutations are listed in Table 1. Eight genes showed at least two such variants, including *F5* (four variants), CR1, AKAP1 (three variants each), ANKRD5, C15orf42, KIF7, ERBB2 and ABCA10 (two variants each). Next, we asked whether the variants have already been annotated in the GWAS catalogue, meaning that they have been already discovered in larger case / control studies. Altogether, 13 different SNPs are listed in the GWAS catalogue as related to glioblastoma. Interestingly, none of the 85 reported variants is associated with GBM. Beyond the effect of single variants on tumor development, the clustering of SNVs in single genes may also contribute to the generation of tumors. To address this issue, we calculated scores for each gene separately and performed a filtering to discover genes with most variants. We also investigated whether the 85 SNVs in 73 genes accumulate at specific genomic positions or on certain molecular pathways.

**Table 1:** Overview of the variants that are homozygous in the children's germlines and heterozygous in the parents' germlines

Chr	Position	rs ID	REF	ALT	Gene	# SNVs
chr1	1334519	rs114112990	G	C	CCNL2	1
chr1	1354515	rs904589	C	G	ANKRD65	1
chr1	19181015	rs34447754	G	C	TAS1R2	1
chr1	20977000	rs1043424	A	C	PINK1	1
chr1	168013850	rs11558511	T	C	DCAF6	1
chr1	169498975	rs6030	T	C	F5	4
chr1	169511555	rs6032	T	C		
chr1	169511734	rs4525	T	C		
chr1	169511755	rs4524	T	C		
chr1	175092707	rs10798333	C	T	TNN	1
chr1	196642233	rs800292	G	A	CFH	1
chr1	200635550	rs3795634	T	C	DDX59	1
chr1	201166383	rs4915221	G	A	IGFN1	1
chr1	203152801	rs880633	T	C	CHI3L1	1
chr1	207753621	rs2274567	A	G	CR1	3
chr1	207782931	rs6691117	A	G		
chr1	207790088	rs3811381	C	G		
chr1	247615261	NA	GA	G	OR2B11	1
chr1	248020556	rs11204523	G	C	TRIM58	1
chr2	10262920	rs1130609	T	G	RRM2	1
chr2	71212129	rs3796100	A	T	ANKRD53	2
chr2	71212405	rs61732279	T	C		
chr2	85622059	rs6886	T	C	CAPG	1
chr2	86400824	rs1050301	G	A	IMMT	1
chr2	88472791	rs4129190	G	A	THNSL2	1
chr2	209190632	rs999890	T	G	PIKFYVE	1
chr2	228102723	rs13424243	G	C	COL4A3	1
chr3	4508742	rs2819590	C	T	SUMF1	1
chr5	122718736	rs6595440	G	C	CEP120	1
chr5	141059158	rs1031904	C	G	ARAP3	1
chr5	149001551	rs4629585	A	C	ARHGEF37	1
chr5	149772280	rs1136103	C	G	TCOF1	1
chr5	150886882	rs1105168	G	A	FAT2	1
chr5	180582604	rs2546423	A	G	OR2V2	1
chr6	12124587	rs2228212	C	G	HIVEP1	1
chr7	6026988	rs1805321	G	A	PMS2	1
chr7	87564497	rs2279542	C	G	ADAM22	1
chr7	88424115	rs2373396	C	G	C7orf62	1
chr8	72975801	rs7819749	T	G	TRPA1	1
chr8	133975283	rs2069561	G	A	TG	1
chr11	3681519	rs2280134	T	C	ART1	1
chr11	5718517	rs7935564	G	A	TRIM22	1
chr11	18743180	rs10832975	C	G	IGSF22	1
chr11	60776209	rs11230563	C	T	CD6	1
chr11	60893235	rs2229177	C	T	CD5	1
chr11	62863518	rs7113279	A	G	SLC22A24	1
chr11	66083129	rs3741367	T	C	CD248	1
chr11	69063393	rs7103126	T	C	MYEOV	1
chr11	93457532	rs78544176	C	G	KIAA1731	1
chr12	18435452	rs11044004	C	T	PIK3C2G	1
chr12	25243115	rs1908946	G	C	LRMP	1
chr12	29604392	rs1347570	C	G	OVCH1	1
chr14	21500121	rs9624	G	T	TPPP2	1
chr14	21796784	rs3748361	G	C	RPGRIP1	1
chr14	76156609	rs2303345	C	T	TTLL5	1
chr14	88651962	rs17762463	C	T	KCNK10	1
chr14	91636532	rs4900072	C	T	C14orf159	1
chr15	78466127	rs2304824	T	C	ACSBG1	1
chr15	89398407	rs3743398	C	T	ACAN	1
chr15	90126121	rs10775247	C	T	C15orf42	2
chr15	90128966	rs11629584	C	T		
chr15	90174824	rs12900805	C	T	KIF7	2
chr15	90176073	rs3803530	C	A		
chr16	71483497	rs72795864	C	G	ZNF23	1
chr16	88504850	rs1105066	G	C	ZNF469	1
chr16	89350038	rs2279348	G	A	ANKRD11	1
chr17	37814080	rs1877031	G	A	STARD3	1
chr17	37879588	rs1136201	A	G	ERBB2	2
chr17	37884037	rs61552325	C	G		
chr17	55182878	rs17761023	C	T	AKAP1	3
chr17	55183792	rs35359994	G	A		
chr17	55183813	rs34535433	A	G		
chr17	66538239	rs2302234	G	T	FAM20A	1
chr17	67125840	rs4968839	C	T	ABCA6	1
chr17	67178316	rs4968849	A	G	ABCA10	2
chr17	67212423	rs9909216	G	A		
chr17	76528790	rs11651537	A	G	DNAH17	1
chr18	6997818	rs12961939	A	C	LAMA1	1
chr21	37617630	rs4817788	T	G	DOPEY2	1
chr22	26222454	rs9624909	C	T	MYO18B	1
chr22	29885016	rs59371099	G	A	NEFH	1
chr22	30762140	rs740223	G	A	CCDC157	1
chr22	30776095	rs5749088	C	T	RNF215	1
chr22	31491295	rs3205187	G	C	SMTN	1
chr22	36537763	rs61741884	C	T	APOL3	1

### Cumulative scores of genes

**Figure 2:**
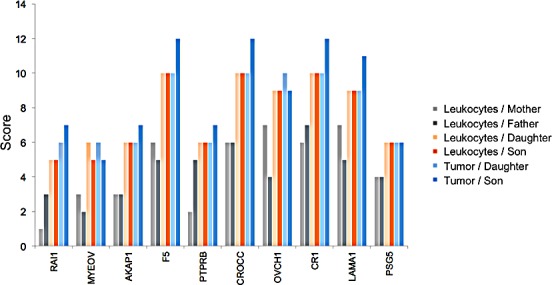
Genes with SNV accumulation The 6 bar charts show for 10 genes how many homozygous and heterozygous variants can be found in the leukocytes of the parents (two leftmost bars per gene), in the leukocytes of the siblings (two middle bars per gene), and in the GBM DNA of the siblings (two right bars per gene). The bar height corresponds to the computed cumulative score.

The variants listed in Table 1 indicate that certain genes may be more affected than others. In this analysis we just considered variants that were homozygous in the siblings and heterozygous in parents. Nonetheless, variants that are heterogeneous may also add to the pathogenicity. To acknowledge this fact, we defined a score for each gene: Each genomic position that is homozygous wild type obtains a score of 0, heterozygous variants a score of 1 and homozygous variants a score of 2. The score of a gene corresponds to the sum over all positions. To find the most affected genes and to trade off between sensitivity and specificity we carried out three filter steps: First, all genes with a score below 5 in children were excluded, leaving the more frequently affected genes. Second, the average score of children has to be at least 150% of the average score of parents, leaving the genes where children are more affected. This analysis however also contained genes where one parent was as affected as one of the children. Third, scores of both children combined had to be higher than the scores of both parents combined. The filtering finally resulted in a set of 10 genes that are summarized together with their scores in Figure 2. Supplemental Table 1 summarizes all high scoring genes. In Figure 2 we show for each gene 6 bars representing the scores of both parents' leukocyte DNA, the scores of the siblings' leukocyte DNA samples and the scores of two GBM DNA samples. Out of the 10 genes, 6 have already been discovered in the initial analysis of homozygous variants in the siblings, namely MYEOV, AKAP1, F5, OVCH1, CR1 and LAMA1. Beyond these, we detected RAI1, PTPRB, CROCC and PSG5.

**Figure 3:**
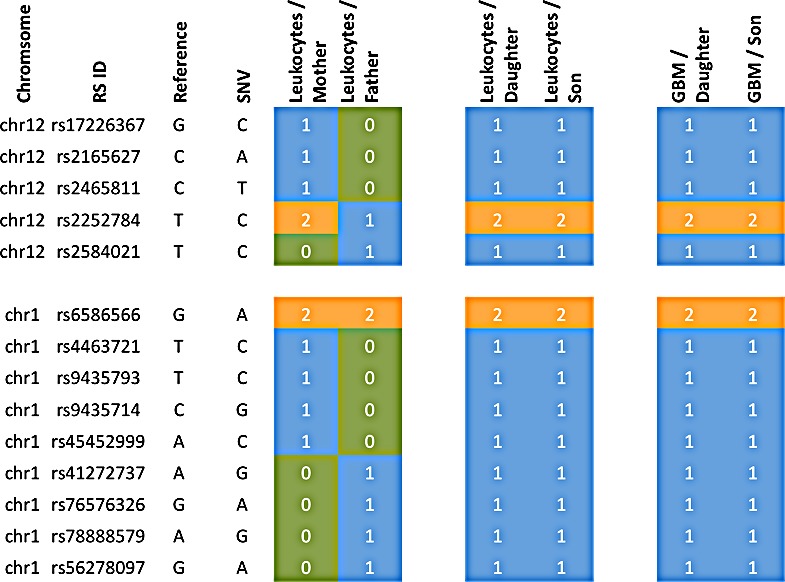
Accumulation of variants from father and mother in the siblings Blue colored genotypes are heterozygous (“1”), green colored genotypes wild type (“0”) and orange genotypes homozygous variants (“2”). The top part represents the gene PTPRB, the bottom part CROCC.

The variants leading to these scores are exemplarily shown for CROCC and PTPRB in Figure 3, for the remaining genes respective material is available in Supplemental Table 2. In this figure it can be seen that the children in both cases have all mutations of both parents, indicating a potential cumulative effect.

**Figure 4:**
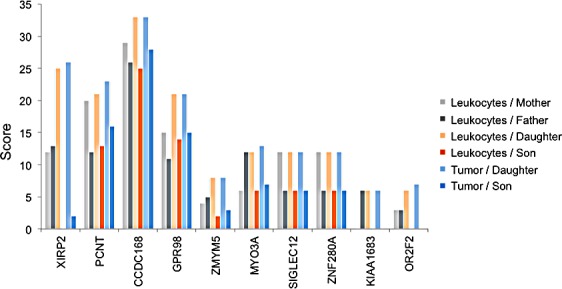
Genes with SNV accumulation in the daughter The 6 bar charts show for 10 genes that are substantially more affected in the daughter as compared to the son how many homozygous and heterozygous variants can be found in leukocytes of parents (two leftmost bars per gene), leukocytes of siblings (two middle bars per gene), and GBM DNA of the siblings (two right bars per gene). The bar height corresponds to the computed cumulative score.

**Figure 5:**
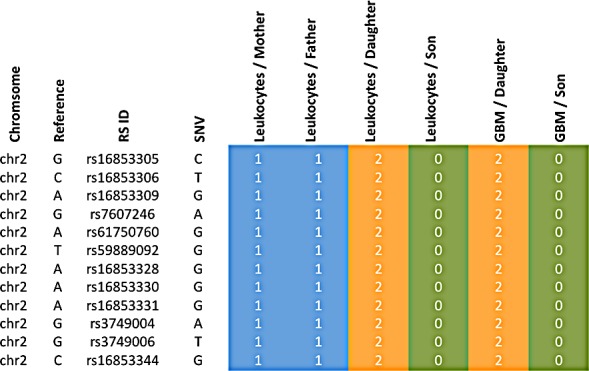
Accumulation of variants for the XIRP2 gene of the daughter Blue colored genotypes are heterozygous (“1”), green colored genotypes wild type (“0”) and orange genotypes homozygous variants (“2”).

Since the daughter developed four different tumors, specific genetic factors may have contributed to the tumorigenesis in the daughter as compared to the son. We carried out the same scoring as described above, but with focus on the sex related differences. We specifically searched for genes that were more affected in the daughter as compared to the son by calculating the difference of the scores (Figure 4). For the gene XIRP2 we obtained scores of 12 and 13 for the mother and the father, respectively, a score of 25 for the daughter and a score of 0 for the son (Figure 5). For the gene PCNT we found a score of 20 and 12 for mother and father, respectively, a score of 21 for the daughter and of 13 for the son. Likewise we found for the daughter an elevated score for the G-protein coupled receptor 98 (GPR98). For this gene, the parents' scores were 15 and 11, the score of the son 13 and the score of the daughter 21 (Supplemental Table 1 provides a summary). The SNV accumulation for the XIRP2 locus with RS ID is given in Figure 5. The according information for other relevant genes is given in Supplemental Table 2. For some genes we also found scores that were elevated for the son as compared to his sister (see Supplemental Tables 1 and 2).

### Statistical enrichment analysis

After interpreting single variants and accumulation on genes we carried out a statistical enrichment analysis to improve the understanding of the variants and affected genes on a systematic level. Specifically, we applied our Gene Set Analysis Tool GeneTrail, as described in the Materials and Methods section. First, we asked whether the 73 genes carrying at least one homozygous variant in the children that are heterozygous in the parents are clustered with respect to the genomic localization. On the highest level we discovered significant clustering on three chromosomes, chromosome 1 (15 genes, adjusted p-value of 2.2*10^−4^), chromosome 22 (7 genes, adjusted p-value of 7.1*10^−4^) and chromosome 11 (10 genes, adjusted p-value of 1.9*10^−3^). Altogether, 32 of all 75 genes have been localized on these three chromosomes. More precisely, we discovered significant enrichment of 12 chromosomal bands. The highest accumulation was calculated for chr22 q12.2 (4 genes, adjusted p-value of 1.6*10^−4^) and chr1 p36.33 (3 genes, adjusted p-value of 3.5*10^−3^). All significant genomic clusters are available in the Supplemental Table 3.

To identify enriched regulatory/metabolic pathways for the genes we applied a KEGG pathway analysis. Here, we found 3 significant pathways that contain at least three affected genes: focal adhesion (4 genes, adjusted p-value of 9.8*10^−3^), ECM-receptor interaction (3 genes, adjusted p-value of 9.8*10^−3^), and complement and coagulation cascades (3 genes, adjusted p-value of 9.8*10^−3^). In the first two paths the genes TNN, LAMA1, ERBB2 and COL4A3 were found, in the complement and conjugate cascades CFH, CR1 and F5. Remarkably, CR1 and F5 belong to the genes with most variants overall (see Table 1).

**Figure 6:**
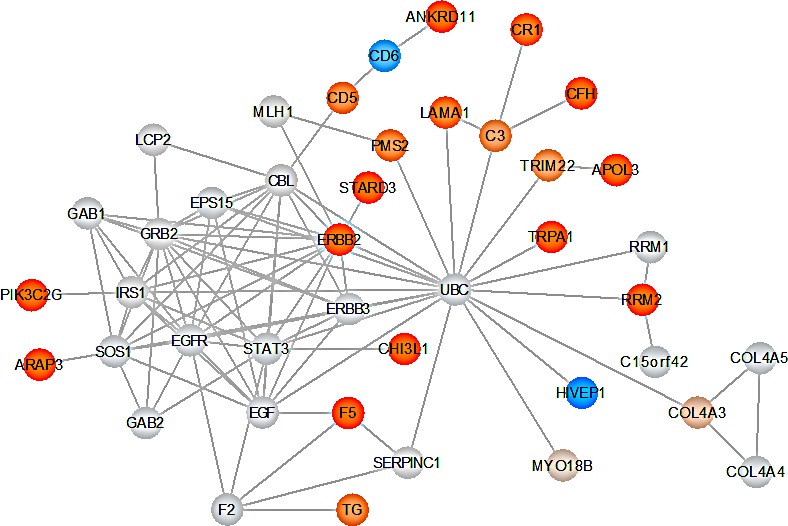
Key GBM network The graphic shows the core-connected component of the interaction network derived from the STRING database. Nodes are colored with respect to the fold change of the score in children and parents. Genes with elevated scores are indicated in orange. Increased color intensity indicates an increased SNV accumulation in the respective gene. Genes with low scores are indicated in blue. Genes indicated in gray are not scored but added by STRING to the network.

The KEGG pathway analysis has two essential drawbacks: pathways are considered as separate entities and show partially a high redundancy. We thus searched for functional interrelations between the affected genes without paying attention to the pre-defined pathways annotated by KEGG. Specifically, we used the STRING database [19] to extract interactions between all genes. Altogether, 100 such interactions have been found by adding 20 additional partner genes. The interactions together with all scores are provided in Supplemental Table 4. The core connected component generated by STRING has been visualized by Cytoscape [20]. To indicate how much the respective parts are affected, the fold change of scores for each gene as computed in the last paragraph has been used to color the nodes. As indicated in Figure 6, several genes including e.g. *F5*, *LAMA1*, *ERBB2* or *STARD3* seems to be in proximity to genes of the EGF/EGFR signaling cascade, which is known to be important in glioma [21].

**Figure 7:**
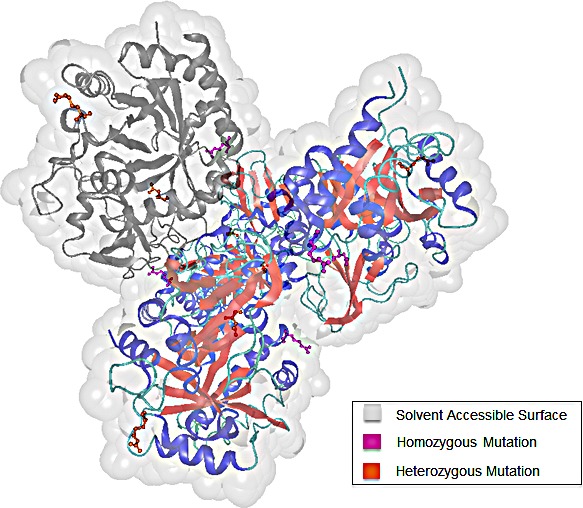
3D structure of chitinase-3-like protein 1 (CHI3L1) The four chains of CHI3L1 are colored according to their secondary structure elements. To highlight the distribution of the detected mutations within one chain, chain C is colored in grey. We differentiate between homozygous (pink) and heterozygous (orange) mutations.

Former studies revealed chitinase 3-like protein 1 (CHI3LI) to be highly expressed in human glioma tissue and hence, to play an important role in the regulation of malignant transformation and local invasiveness in gliomas [22]. Figure 7 displays the 3D structure of CHI3LI with highlighted mutations detected within our data set. The representation was created via the molecular modeling visualization tool BALLView [23].

### Mutations in tumor DNA not present in leukocytes

So far we mainly focused on germline mutations. To gain further insights into the genetic alterations of the tumor during its development, we performed a second analysis filtering those variants that became homozygous in tumor while being heterozygous or wild-type in the children's leukocyte DNA. Just one single variant showed the required genotype composition in leukocyte and tumor DNA, a frameshift mutation in the gene RAI1. This gene has already been discovered to be more affected in the siblings' leukocyte DNA as compared to parental leukocytes. Next, we searched for variants that were homozygous wild type in leukocyte DNA but heterozygous in tumor DNA of both siblings. Altogether we discovered 15 such variants that are summarized in Table 2. Among them we found variations in genes *TP53* (rs121913343), *LOXL3* (chr2:74763923, G>GC), *CSPG4* (rs79463888), and *ARID1B* (chr6:157528243, C>T).

**Table 2:** SNVs in tumor DNA of the siblings that were not present in their leukocyte DNA

CHROM	POS	rs ID	REF	ALT	Gene
chr1	3389970	.	G	A	ARHGEF16
chr2	74763923	.	G	GC	LOXL3
chr6	151815279	rs199768731	A	C	CCDC170
chr6	157528243	.	C	T	ARID1B
chr7	22184668	.	G	A	RAPGEF5
chr11	89883678	.	G	A	NAALAD2
chr15	75982085	rs79463888	C	T	CSPG4
chr16	3025782	.	G	A	PKMYT1
chr16	30750387	.	G	A	SRCAP
chr16	85682289	.	A	AC	KIAA0182
chr17	7577121	rs121913343	G	A	TP53
chr17	77111776	.	C	G	RBFOX3
chr19	6374295	.	G	A	ALKBH7
chr19	54649413	.	G	A	CNOT3
chr20	60775922	rs35693261	C	T	GTPBP5

## DISCUSSION

Familial exome sequencing is a valuable tool to unravel associations between the genotype and pathogenic processes. Respective studies are not only relevant for Mendelian disorders but can also be applied to diseases with complex hereditary factors. In this study, we described the whole exome analysis of a family with two unaffected parents and two siblings suffering of glioblastoma multiforme (GBM) at an early age. Considering the multiple incidences of different carcinoma types in the family pedigree and the unusual early development of GBM in both children, we assumed a genetic predisposition promoting the GBM development. To confirm this hypothesis, we concentrated especially on non-synonymous variants that became newly homozygous in both children while being heterozygous in both parents. In total, we detected 85 such variants in 73 different genes, containing previously known key players in GBM tumorigenesis such as PMS2. To reflect the complexity and molecular heterogeneity of GBM we calculated accumulation of variants in genes and performed a pathway and network analysis to get an overview on the interplay of the identified genes.

In addition to the accumulation of variants in genes we also performed statistical enrichment analysis. The results of our network analysis present several key players that seem to be relevant for the tumorigenesis of GBM. Polymorphisms in *CR1* (*CD35*) are discussed to play a role in cerebral amyloid angiopathy, which is a leading cause in intracerebral hemorrhage [24] and might even be associated with the risk of Alzheimer's disease [25]. Notably, the factor V Leiden mutation [26], which is a common risk factor for thrombophilia, is a known variant of *F5* that is a member of the complement and coagulation cascades like *CR1*. Although, we did not find the factor V Leiden mutation, the *F5* gene accumulated the most new non-synonymous homozygous variants in both children. These homozygous variants might also impact the F5 protein function. The interplay of intracerebral hemorrhage and thrombophilia may cause microenvironments of oxygen deprivation / hypoxia promoting the development of GBM. Hypoxia has been described as an important pathogenic factor in GBM and other malignancies [27, 28].

Another interesting group of genes are *TNN*, *LAMA1*, and *ERBB2*, where all three are involved in focal adhesion, and the first two additionally in the ECM-receptor interaction pathway. Those two pathways are crucial for the migration of cells and for the maintenance of tissue architecture [29]. Furthermore, these processes contribute to tumor invasion of the surrounding normal tissues. Especially ERBB2, being the dimerization partner of EGFR, has previously been associated with glioma risk. Previous SNP analysis of glioma patients indicated *ERBB2* as a low penetrance gene associated with risk of glioblastoma development [30]. The SNP rs1058808 in *ERBB2* mentioned in the publication of Andersson *et al.* [30] corresponds to the variant that we described, which became homozygous in both siblings. We also found a heterozygous variant in *EGFR* (chr7:55229255, rs2227983, G>A) for father and son. The increase of the non-synonymous homozygous SNV load in both children's leukocyte DNA in genes related to the EGFR signaling cascade likely contributes to an increased risk/predisposition for GBM in both siblings.

In the neighborhood of these genes we discovered a strong association with GBM for the gene PMS2, an important gene for DNA repair. PMS2 has been demonstrated to be important for GBM in various studies [31, 32]. Most interestingly, Walter and co-workers describe the case of a 13-year-old child presented with three simultaneous malignancies, including GBM. Here, the genetic analysis also revealed a homozygous mutation in the PMS2 gene [33].

Remarkably we also discovered a mutation in TP53 in GBM DNA of siblings, which was not present in leukocytes. Altogether, 15 genes showed respective genotype composition, including *ARID1B,* a component of the SWI/SNF chromatin remodeling complex also relevant for the chromatin architecture [34, 35]. Expression of the transmembrane proteoglycan CSPG4 has been associated with melanoma formation and takes place in a number of normal tissues throughout development [36]. Expression of CSPG4 seems to correlate with poor prognosis in several cancer types including GBM [37]. *LOXL3* encodes a member of the lysyl oxidase gene family. Members of this family are responsible for the development of cross-links in extracellular matrix proteins, such as collagens and elastin [38], and may play a role in tumor progression [39].

In addition, the development of tumors in both siblings at an early age indicates that some of the genes, which have been identified in our SNP analysis but not yet been associated with GBM, may also play a role the development of GBM.

Since the daughter was diagnosed with four different tumors, we also investigated gender specific changes. For the daughter we found an accumulation of variants for several genes including XIRP2, PCNT, CCDC168 and GPR98, the latter of which was reported to be associated with GBM survival by alternative exon usage [40]. Notably, exon level analysis recently defined several new GC-regulated transcript variants including GPR98 in childhood acute lymphoblastic leukemia [41]. The gene XIRP2 that was most affected in our analysis, was reported to be expressed mainly in striated muscles [42], but it has not yet been connected to tumor development.

In a previous study we searched for SNPs in glioblastoma samples by targeted re-sequencing [43]. Interestingly, several of the identified genes including MDM1, PAXIP1, PARP1, SART1 and B4GALNT1, showed also mutations in the present study. These genes were, however, not affected in both children but predominantly in the son.

## CONCLUSIONS

In summary, we have identified a group of genes all of which show an accumulation of homozygous or heterozygous germline variants in both siblings. Especially the daughter, who was diagnosed with four different tumors, seems to be strongly affected. By using prediction tools for regulatory pathways and functional interaction information we showed that these genes are likely involved in specific pathways including focal adhesion and ECM receptor interaction, which are known to be relevant for GBM development. The unfortunate accumulation of homozygous variants in both siblings might have promoted the early onset of GBM in both children with both carrier parents unaffected.

## MATERIAL AND METHODS

### Samples

We collected Glioblastoma multiforme (GBM) tumor samples from two children, brother and sister, that came down with the diagnosis at the age of 10 and 12 years. The sister suffered from a supratentorial GBM (WHO grade IV) and from a pilocytic infratentorial astrocytoma (WHO grade I) and the brother from a GBM (WHO grade IV), based on the right ventricular trigonum with thalamic infiltration of the dorsal ventricular structures, of the septum pellucidum and the midbrain. The sister also developed a common acute lymphatic leukemia (cALL) 8 months after the diagnosis of GBM as well as a diffuse pontine glioma at the age of 15 years. We also collected whole blood from the children in lithium heparin plasma tubes. Both parents are healthy without any history of brain tumor or cancer disease. In the history of the family, we found from the paternal side, two cases of meningioma that did not require any treatment, one case of ovarian carcinoma and one case of colon carcinoma. From the maternal side, we found one case of prostate carcinoma and one case of melanoma. To determine whether the GBMs of the children were caused by a genetic anomaly inherited from the parents, we also collected whole blood in lithium heparin tubes from the parents to analyze differences and similarities between the exomes of the leukocytes from the children and the leukocytes from the parents additional to the comparison of the exomes of both children's tumors. All samples were obtained with parents' informed consent and the local ethic committee (“Ethik-Kommission der Ärztekammer des Saarlandes”, No. 67/06) approved the study. The isolation of genomic DNA from the GBM tumor samples of both children and from the parents' and children's leukocytes was performed according to standard protocols. For tumor DNA isolation, five 10μm tumor slices have been used. Adjacent slices have been histologically confirmed to have tumor cell content above 90%.

### Exome sequencing and bioinformatic analyses

We performed whole exome capture and sequencing for a total of six samples consisting of blood samples of all four individuals, the healthy parents and the two affected children, as well as DNA from the glioblastoma of the latter two. The six samples were enriched using Illumina'sTruSeq Exome Enrichment Kit, targeting 62 Mb of exomic sequence, including 5′-UTR, 3′-UTR, microRNA, and other non-coding RNA. Exome sequencing of 2×100 bp paired-end reads was performed for all six samples together on one lane of an Illumina HiSeq2000. The reads were mapped against the human reference genome build hg19 using BWA [12], followed by the removal of PCR duplicates with Picard (http://picard.sourceforge.net).

Following the Genome Analysis Toolkit (GATK) Best Practices v3 guideline, we used the GATK 1.6-11 [13] for further processing the mapped reads and for SNP and indel calling. In brief, we computed the intervals for local realignment and performed the realignment to reduce false positive variant calls around indels. After fixing the mate information for paired-end reads, we performed the GATK quality score recalibration steps. Finally, the SNPs/indels were called for the six input samples simultaneously with the GATK UnifiedGenotyper using the following default parameters: -stand_call_conf 50.0 -stand_emit_conf 10.0. After that we applied the proposed filtering for small sample exome data using VariantFiltration.

For annotating the genes and class of SNPs/indels, we used ANNOVAR [14]. Next, we applied different filters. In order to detect potential relevant SNVs we identified variants where the parents were heterozygous and both siblings homozygous for the variant. Since single variants may not suffice to represent the heterogeneous genetics of GBM we searched for an accumulation of effects. First, clustering of affected genes on pathways and genomics positions was investigated. Here, the corresponding genes identified in the previous step were analyzed with respect to their enrichment in KEGG pathways [15, 16] and chromosome bands with GeneTrail [17]. The computed p-values for these categories were FDR adjusted [18] and considered significant if smaller than 0.05. Beyond the pathway analysis we also searched for accumulation of variants in genes, i.e. cases where both parents carry homozygous or heterozygous variants that finally accumulate in both siblings.

To construct a complex network we used STRING using the standard parameters, i.e. we included Neighborhood, Gene Fusion, Co-occurrence, Co-expression, Experiments, Databases and Textmining [19]. To augment the network we used twice the “add” option and thereby added 20 additional partners. The resulting networks with scores are provided in the Supplemental Material of this manuscript.

## SUPPLEMENTARY MATERIAL AND TABLES








